# Ayurveda for Managing Noncommunicable Diseases in Organisation for Economic Cooperation and Development Nations: A Qualitative Systematic Review

**DOI:** 10.1002/hsr2.70624

**Published:** 2025-04-08

**Authors:** Patricia Egwumba, Haiquan Wang, Laura Nellums, Manpreet Bains, Kaushik Chattopadhyay

**Affiliations:** ^1^ Lifespan and Population Health, School of Medicine University of Nottingham Nottingham UK; ^2^ School of Exercise and Health Shanghai University of Sport Shanghai China; ^3^ The Nottingham Centre for Evidence‐Based Healthcare: A JBI Centre of Excellence Nottingham UK; ^4^ Health Sciences Centre, College of Population Health University of New Mexico Albuquerque New Mexico USA

**Keywords:** Ayurveda, management, noncommunicable diseases, Organisation for Economic Cooperation and Development, qualitative systematic review

## Abstract

**Background:**

Ayurveda, a traditional system of medicine, has gained recognition in the Organisation for Economic Cooperation and Development (OECD) countries as a complementary and alternative medicine for managing noncommunicable diseases (NCDs). Qualitative studies have been conducted in various OECD countries regarding the use of Ayurveda for NCD management. However, no qualitative systematic review has been conducted on this topic.

**Aim:**

This review aimed to synthesize the experiences, perceptions, and perspectives of patients with NCDs and Ayurvedic practitioners on the use of Ayurveda for NCD management in OECD countries.

**Methods:**

The JBI qualitative systematic review guidelines were followed. Several databases were searched to identify published and unpublished qualitative studies.

**Results:**

Of the 18,541 records identified, 9 studies met the eligibility criteria and were included in the review. Using the JBI checklist for qualitative research (10 criteria), the critical appraisal scores of the studies ranged from moderate to high quality. Patients turned to Ayurveda because of concerns about side effects and dissatisfaction with conventional Western treatments and were driven by the perceived gentleness and holistic qualities of Ayurveda. Complementing these patient insights, Ayurvedic practitioners emphasized that Ayurveda identifies and addresses the root causes of diseases rather than treating symptoms alone. Integration challenges, limited medication access, and regulatory constraints were identified as factors affecting Ayurveda's service delivery.

**Conclusions:**

Patients preferred Ayurveda because of its natural approach and fewer side effects, whereas Ayurvedic practitioners valued its holistic approach. However, its wider acceptance has been hampered by hurdles such as regulatory barriers and limited access to medicines. Strategies to overcome some of the barriers identified in this review as well as to promote the strengths discussed in this review may facilitate the effective use of Ayurveda to manage NCDs in OECD countries.

**Trial Registration:** PROSPERO, Registration No. CRD42023397952.

## 1 Introduction

The growing burden of noncommunicable diseases (NCDs) and increasing healthcare expenditure are primary challenges and concerns for the Organisation for Economic Cooperation and Development (OECD) countries [1, 2, 3, 4]. In 2019, NCDs were the leading cause of morbidity and the greatest contributor to low life expectancy in several OECD countries [5]. Among OECD member countries, NCDs accounted for 88% of all years lived with disability, 85% of all disability‐adjusted life years, and 89% of all deaths [6]. Over the years, the OECD has played a crucial role in promoting collaboration among member countries to tackle NCDs effectively [5]. Up until now, Western medicine has been the mainstay of NCD care in many OECD countries [7]; however, some patients refused to remain on lifelong Western medications because of perceived drug interactions and side effects [8]. Such apprehensions have compelled some NCD patients to explore alternative ways to manage their health conditions [8].

Ayurveda is an ancient traditional system of medicine that originated in the Indian subcontinent more than 5000 years ago [9]. In Ayurveda, detoxifying and purifying therapies such as Panchakarma and medicines containing plant‐, animal‐, or mineral‐origin ingredients are mainly used [10]. Ayurveda has been increasingly accepted and recognized as an alternative medicine in major countries across the OECD [11]. Qualitative studies have been conducted among patients and Ayurvedic practitioners in various OECD countries regarding the use of Ayurveda for NCD management [12, 13, 14, 15], however, to date, no qualitative systematic review on this topic has been conducted. Therefore, this systematic review aimed to synthesize the experiences, perceptions, and perspectives regarding the use of Ayurveda for NCD management. OECD countries often have robust systems for developing and implementing policies, including on health and social care [5]. Thus, the review findings would help make recommendations for research, policy, and practice.

## 2 Methods

This systematic review was conducted according to a published protocol [16]. The protocol was also registered with PROSPERO (CRD42023397952).

### 2.1 Inclusion Criteria

#### 2.1.1 Participants

This review included studies that were conducted among patients (aged 18 years and older) with one or more NCDs or Ayurvedic practitioners who managed NCDs and resided in any OECD member country.

#### 2.1.2 Phenomena of Interest

This review included studies that explored experiences, perceptions, and perspectives regarding the use of Ayurveda for managing NCDs.

#### 2.1.3 Context

The review included studies that were conducted in any OECD member country: Australia, Austria, Belgium, Canada, Chile, Colombia, Costa Rica, Czech Republic, Denmark, Estonia, Finland, France, Germany, Greece, Hungary, Iceland, Ireland, Israel, Italy, Japan, Korea, Latvia, Lithuania, Luxembourg, Mexico, the Netherlands, New Zealand, Norway, Poland, Portugal, Slovakia, Slovenia, Spain, Sweden, Switzerland, Turkey, the UK, and USA [17]. Any study setting was eligible, such as community, primary care, secondary care, and tertiary care.

#### 2.1.4 Types of Studies

This review included qualitative studies with designs such as phenomenology, ethnography, grounded theory, narrative research, case studies, and action research and data collection methods such as semi‐structured interviews, focus group discussions, observations, documents, case note analyses, and diaries. In addition, mixed‐methods studies that reported qualitative data were eligible.

### 2.2 Search Strategy

The following electronic databases were searched from their inception dates until January 30, 2024, to locate published studies: (i) MEDLINE (Ovid), (ii) Embase (Ovid), (iii) CINAHL (EBSCOhost), (iv) PsycINFO (Ovid), (v) AMED (Ovid), and (vi) Web of Science. Additionally, EThOS (British Library) and ProQuest Dissertations and Theses were searched for unpublished studies. The search strategies, initially developed for MEDLINE, were later adapted for other databases and are detailed in Appendix SA. The search strategies were developed in consultation with an experienced research librarian at the University of Nottingham (UK). The terms for “Ayurveda” and “qualitative” were based on search strategies from previous systematic reviews [18, 19, 20]. No language restrictions were applied. The reference lists of all included studies were also screened for additional relevant studies.

### 2.3 Study Selection

Following the search, all identified citations were collated and uploaded into EndNote X9, and duplicate citations were removed [21]. The titles and abstracts were screened for eligibility by two independent reviewers (P. E. and H. W.) using the inclusion criteria. Both were trained JBI systematic reviewers. Studies identified as potentially eligible or those without an abstract were retrieved, and their full text was imported into Rayyan [22]. The full texts of the studies were assessed against the inclusion criteria by the two independent reviewers (P. E. and H. W.). Full‐text studies that did not meet the inclusion criteria were excluded. Disagreements were resolved through discussion between the reviewers, and a third reviewer was not required.

### 2.4 Assessment of Methodological Quality

All studies selected for inclusion were critically assessed for methodological quality by the two independent reviewers (P. E. and H. W.) using the JBI critical appraisal checklist for qualitative research [23]. This checklist uses a series of criteria that are scored as being met, not met, unclear, or not applicable to the particular study. Disagreements were resolved through discussion between the reviewers, and a third reviewer was not required. Data extraction and synthesis were performed for all studies that met the inclusion criteria regardless of methodological quality.

### 2.5 Data Extraction

Data were extracted from the included papers by the two independent reviewers (P. E. and H. W.) using an adapted JBI data extraction tool. The reviewers extracted the following study characteristics: author, year of publication, participant characteristics, OECD country, study setting, study design, data collection methods, and data analysis technique. Findings relevant to the review question and specific objectives were extracted. The two reviewers (P. E. and H. W.) assigned levels of credibility to each of the findings using the three levels described by the standardized JBI qualitative data extraction tool: (i) Unequivocal (U): findings accompanied by an illustration beyond reasonable doubt and therefore not open to challenge; (ii) Credible (C): findings accompanied by an illustration lacking clear association with it and therefore open to challenge; and (iii) Unsupported (US): findings were not supported by the data. These were then discussed among the two reviewers (P. E. and H. W.), resulting in a consensus regarding the allocation of these levels [24].

### 2.6 Data Synthesis

Study findings from all qualitative studies were pooled using the JBI system for the unified management, assessment, and review of information (JBI SUMARI) [16], using a meta‐aggregation approach [23]. This involved the aggregation of findings to generate a set of statements representing that aggregation by assembling the findings and categorizing them based on similarity in meaning [24]. These categories were then subjected to synthesis to produce a single comprehensive set of findings [24]. To identify any associated differences or similarities in experiences, perceptions, and perspectives regarding the use of Ayurveda for managing NCDs, data from patients and Ayurvedic practitioners were synthesized separately. Categories of the synthesized findings and accompanying descriptions were created using the words and terminologies used by the participants in the illustrations. These were discussed among the review team and were revised until a consensus was reached before finalization.

## 3 Results

### 3.1 Study Inclusion

**Figure 1:**
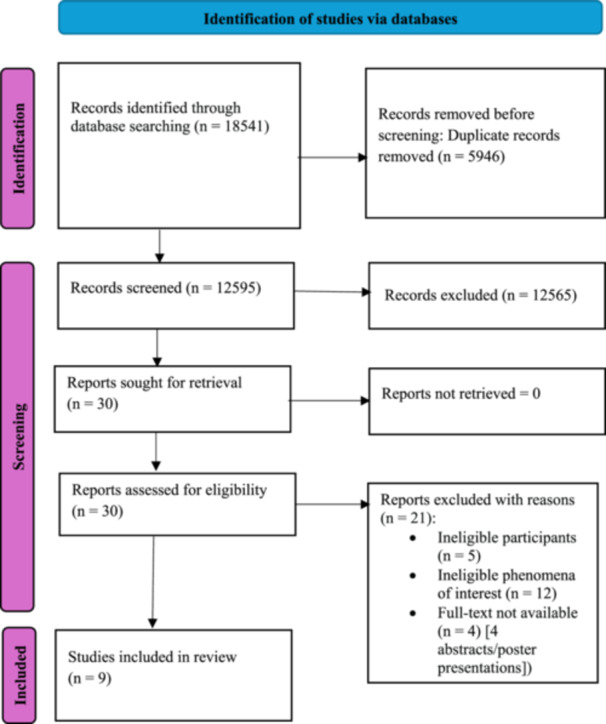
PRISMA flow diagram for included studies from searches of databases [30].

A total of 18,541 records were identified through database searches. After duplicate removal, the titles and abstracts of the remaining records were assessed (*n* = 12,595); a further 12,565 of these records were excluded, leaving 30 papers eligible for full‐text screening. The reasons for the exclusions are presented in Appendix SB. Nine studies [12, 13, 14, 15, 25, 26, 27, 28, 29] were eligible for inclusion in the review. Figure 1 illustrates the study selection process.

### 3.2 Methodological Quality

The critical appraisal results of the included studies are presented in Table 1 [12, 13, 14, 15, 25, 26, 27, 28, 29]. Eight studies had results of 7 or above out of 10 [12, 13, 14, 15, 25, 26, 27, 29]. Only one study had a score of 6 out of 10 [28]. Six of the 10 quality appraisal questions achieved a high proportion of “yes” ratings; however, Questions 1, 6, 7, and 9 had a significantly lower proportion of “yes” ratings. Six studies excluded statements that identified the researchers' philosophical perspectives [12, 13, 15, 25, 26, 28]. Three authors did not explicitly discuss their cultural or theoretical positioning in their studies, which was crucial for understanding potential biases and assumptions that might have influenced the research process [14, 15, 28]. Four studies did not provide evidence of ethical approval by an appropriate body [13, 26, 28, 29].

**Table 1:** Critical appraisal of included studies (*n* = 9).

References	Q1	Q2	Q3	Q4	Q5	Q6	Q7	Q8	Q9	Q10
Frank and Stollberg [13]	U	Y	Y	Y	Y	Y	N	Y	U	Y
Abraham and Young [25]	U	Y	Y	N	Y	Y	N	Y	Y	Y
Rao and Lyn [26]	U	Y	Y	Y	Y	Y	Y	Y	U	Y
Warrier [28]	U	Y	Y	Y	Y	N	N	Y	U	Y
Wiese and Oster [29]	Y	Y	Y	Y	Y	Y	N	Y	U	Y
Moodley and Oulanova [14]	Y	Y	Y	Y	Y	N	N	Y	Y	Y
Dhruva et al. [15]	U	Y	Y	Y	Y	N	N	Y	Y	Y
Santosh [27]	Y	Y	Y	Y	Y	Y	Y	Y	Y	Y
Niemi and Ståhle [12]	U	Y	Y	Y	Y	Y	Y	Y	Y	Y
% “yes” responses	33	100	100	89	100	67	33	100	56	100

*Note:* Criteria for the critical appraisal of qualitative evidence:

Q1 = Is there congruity between the stated philosophical perspective and the research methodology?

Q2 = Is there congruity between the research methodology and the research question or objectives?

Q3 = Is there congruity between the research methodology and the methods used to collect data?

Q4 = Is there congruity between the research methodology and the representation and analysis of data?

Q5 = Is there congruity between the research methodology and the interpretation of results?

Q6 = Is there a statement locating the researcher culturally or theoretically?

Q7 = Is the influence of the researcher on the research, and vice‐versa, addressed?

Q8 = Are participants, and their voices, adequately represented?

Q9 = Is the research ethical according to current criteria or, for recent studies, and is there evidence of ethical approval by an appropriate body?

Q10 = Do the conclusions drawn in the research report flow from the analysis, or interpretation, of the data?

Abbreviations: N, No; U, Unclear; Y, yes.

### 3.3 Characteristics of the Included Studies

All included studies investigated the perspectives of participants. These studies spanned regions, with one each from Australia [29], Germany [13], and Sweden [12], and two each from Canada [14, 25], the USA [15, 26], and the UK [27, 28]. Of the nine studies, six were conducted in clinic settings [12, 15, 26, 27, 28, 29] and three in communities [13, 14, 25]. Studies were published between 2002 and 2016. Two studies were conducted with patients [12, 13], six with Ayurvedic practitioners [14, 15, 25, 27, 28, 29], and one study was conducted with both patients and Ayurvedic practitioners [26]. Of the seven studies conducted with Ayurvedic practitioners, only two provided detailed information about practitioner qualifications [15, 27]. Both studies included Ayurvedic practitioners having at least a medical degree in Ayurveda (BAMS) and higher degrees (such as MD and PhD in Ayurveda). However, both studies also included Ayurvedic practitioners with other qualifications such as a diploma or MSc in Ayurveda. The characteristics of the included studies are presented in Table 2.

**Table 2:** Characteristics of included studies.

References	Participant characteristics	Country	Setting	Study design	Data collection	Data analysis
Frank and Stollberg [13]	14 patients (aged 30–66 years; 10 female, 4 male; with NCDs like allergies, chronic back pain, rheumatism, skin conditions, Crohn's disease, cancer, eye disease with imminent blindness)	Germany	Community	Qualitative, explorative	Semi‐structured interviews	Cross‐case and individual analysis
Abraham and Young [25]	Five Ayurvedic practitioners (age, sex, qualifications, years of NCD experience not reported)	Canada	Community	Not reported	Semi‐structured interviews	Unstated in the study
Rao and Lyn [26]	Three Ayurvedic practitioners (age, sex, qualifications, years of NCD experience not reported)	United States	Clinic	Explorative‐descriptive case study	Semi‐structured interviews	Long‐table approach
	3 patients (age, sex, NCD details not reported)					
Warrier [28]	Ayurvedic practitioners (sample size, age, sex, qualifications, years of NCD experience not reported)	United Kingdom	Clinic	Not reported	Semi‐structured interviews	Thematic analysis
Wiese and Oster [29]	Three Ayurvedic practitioners (aged 26–69 years; sex, qualifications, years of NCD experience not reported)	Australia	Clinic	Grounded theory	Semi‐structured interviews	Open and selective coding
Moodley and Oulanova [14]	Two Ayurvedic practitioners (with 11–32 years of NCD experience; age, sex, qualifications not reported)	Canada	Community	Grounded theory	Semi‐structured interviews	Grounded theory
Dhruva et al. [15]	Ten Ayurvedic practitioners (qualifications: BAMS, MD Ayurveda, PhD Ayurveda, diploma Ayurveda, MSc Ayurveda; with 9–45 years of NCD experience; age, sex not reported)	United States	Clinic	Qualitative, explorative	Semi‐structured interviews	Thematic analysis
Santosh [27]	Seventeen Ayurvedic practitioners (qualifications: BAMS, MD Ayurveda, PhD Ayurveda, diploma Ayurveda, MSc Ayurveda; age, sex, years of NCD experience not reported)	United Kingdom	Clinic	Grounded theory	Semi‐structured interviews	Thematic analysis
Niemi and Ståhle [12]	Eighteen patients (aged 37–70 years; 12 female, 6 male; with NCDs like/of neoplasms, circulatory system, respiratory system, digestive system, genitourinary system)	Sweden	Clinic	Mixed methods	Semi‐structured interviews	Systematic text condensation

### 3.4 Review Findings

The synthesized findings collated the experiences, perceptions, and perspectives of patients and Ayurvedic practitioners regarding the use of Ayurveda for managing NCDs in OECD countries. After aggregating 61 findings, 13 categories were generated. The 13 categories were grouped into 4 synthesized findings (2 from the perspective of patients and 2 from that of Ayurvedic practitioners). The synthesized findings from the patients were as follows: (i) Reasons for Ayurvedic use (eight unequivocal findings) and (ii) Perceived benefits and challenges of using Ayurveda (eight unequivocal findings and three credible findings). The synthesized findings from the Ayurvedic practitioners were as follows: (i) Ayurvedic approach to NCD management (13 unequivocal findings and 4 credible findings) and (ii) Factors influencing the provision of quality Ayurvedic care (20 unequivocal findings and 5 credible findings). All findings were supported by illustrations from the included papers (see Appendix SC).

### 3.5 Synthesized Findings From Patients' Experiences, Perceptions, and Perspectives

#### 3.5.1 Synthesized Finding 1: Reasons for Ayurvedic Use

This synthesized finding indicated that patients with NCDs often resorted to Ayurveda primarily because of their dissatisfaction with the side effects and effectiveness of conventional Western medicines [12]. Additionally, many patients preferred Ayurveda because they viewed it as a more natural alternative to Western treatments [12, 13]. This synthesized finding was derived from eight findings, which were merged into two categories (see Appendix SC).

##### 3.5.1.1 Category 1.1: Perceived Side Effects of Conventional Western Medicine

Patients reported experiencing various side effects from conventional Western medicines, ranging from minor issues like nausea and vomiting to more severe complications such as liver damage and kidney failure [12, 13]. For example, one study highlighted a patient's experience with asthma medication:

“I used asthma sprays daily for half a year. Six months later, I felt terrible. I vomited day and night … Then I developed an oedema … I read the description of the spray's eventual side effects, and there it was: vomiting and oedema. I thought I would lose it. I felt tricked because I had asked him three times: ‘Couldn't it be because of the spray?’ … I never went to see him again. I told myself, ‘You will not destroy me.’ I had had enough”

[13, p. 228]

This dissatisfaction was further compounded by concerns about the prolonged use and dependency on prescribed Western medications [12]. In contrast, patients perceived Ayurveda as a gentler, more natural, and holistic alternative to Western medical treatments [12, 13]. Those who incorporated Ayurvedic practices into their health regimens reported experiencing no side effects compared to conventional Western treatments [13].

##### 3.5.1.2 Category 1.2: Dissatisfaction With Conventional Western Medical Treatment Outcomes

Patients recounted their dissatisfaction with conventional Western medical treatments, describing how these approaches often failed to provide satisfactory relief [12, 13]. They shared experiences of using Western medicine to treat conditions like eczema, irritable bowel syndrome, and rheumatoid arthritis, yet continued to struggle with persistent pain and inflammation [12, 13]. For instance, in one study, a participant's experience with eczema treatment highlighted this dissatisfaction:

“As it was with my eczema, it was horrible, really all over my body. And there was no one [in biomedical healthcare] who asked me anything really. Not even what I worked with, in case there could be something in my work environment that I was reacting to. I mean there were no questions whatsoever, they were just ‘here you have some cortisone.’ And then I got some antibiotics, because I had scratched my skin broken, so it had gotten infected. But nothing else.”

[12, p. 7]

Despite their efforts, these treatments proved ineffective, leaving them frustrated [12]. When patients voiced concerns about the effectiveness of prescribed medications, they felt dismissed by Western medical practitioners, who asserted that there were no alternative options [13].

#### 3.5.2 Synthesized Finding 2: Perceived Benefits and Challenges of Using Ayurveda

This synthesized finding highlighted the perceived benefits of Ayurveda in relieving symptoms associated with NCDs [12, 13]. It also addressed the challenges patients faced, such as adhering to complex Ayurvedic treatment regimens and the difficulty of integrating these practices into their daily lives [12]. This synthesized finding was derived from 11 findings, which were merged into two main categories (see Appendix SC).

##### 3.5.2.1 Category 2.1: Relief of Symptoms

Patients reported varying degrees of symptom relief after using Ayurvedic treatments [12]. Physical improvements were noted, such as the cessation of muscle pains, disappearance of eczema within 2 weeks, and alleviation of stomach pain [12, 13]. In one study, a patient with rheumatic pain shared her experience:

“I had muscle rheumatism… […] So I started doing as she said, I began with excluding everything… and you know, within a week I was totally pain‐free … Yes, I haven't needed to eat medication since then!”

[12, p. 9]

Additionally, some patients experienced more subtle benefits, like increased bodily awareness and stress reduction [12, 13]. Improvements in mental health were also reported, with one patient noting better sleep and clearer thoughts. This relief led patients to view Ayurveda as a credible medical system, prompting some to question its lack of integration into mainstream healthcare [12].

##### 3.5.2.2 Category 2.2: Challenges in Using Ayurveda

Patients frequently reported that Ayurvedic treatment plans were comprehensive, encompassing recommendations for diet, lifestyle changes, herbs, and holistic health practices [12, 13]. However, some found these recommendations unrealistic due to the significant lifestyle changes and strict adherence required, which were challenging to sustain in modern, fast‐paced lifestyles [12, 13]. As highlighted in one study, a patient explained:

“But actually, it's quite difficult these days, in this society, to apply everything that is recommended at a consultation.”

[12, p. 9]

While some patients observed positive effects from Ayurveda, they noted that these treatments often took longer to produce results, necessitating patience and persistence [12]. The cost of Ayurvedic treatments was also a concern, particularly in regions where they were not covered by health insurance [13]. Additionally, patients reported difficulties in gaining support from family members, friends, and the broader medical community, especially in areas where Ayurveda was not well‐recognized or culturally accepted [12, 13].

### 3.6 Synthesized Findings From Ayurvedic Practitioners' Experiences, Perceptions, and Perspectives

#### 3.6.1 Synthesized Finding 1: Ayurvedic Approach to NCD Management

This synthesized finding elaborated on the Ayurvedic perspectives regarding NCD management. According to Ayurveda, NCDs are a combined result of important parameters such as imbalance in the three Doshas and accumulation of toxins (Ama). This synthesized finding was extracted from 17 findings, which were merged into 5 categories.

##### 3.6.1.1 Category 1.1: Focus on Treating the Root Cause

Practitioners of Ayurvedic medicine believed that the health of an individual is restored with the balance of the three Doshas (Vata, Pitta, and Kapha), digestive fire (Agni), body tissues (Dhatus), and waste products (Malas), whereas disease arises from imbalances in these elements and the accumulation of toxins (Ama) [25, 26]. They claimed that this philosophy significantly differs from the underlying philosophy of conventional Western medicine, which tends to target the symptoms of a condition without addressing the root causes of the disease [26]. This focus on addressing the root cause of the issue is a hallmark of Ayurveda's holistic philosophy. For instance, one practitioner in a study noted:

“Allopathy suppresses symptoms and only offers standardised treatment options. On the other hand, Ayurvedic medicine treats the root cause and holistically treats the individual person through a gentler system, which encourages the body to heal itself.”

[26, p. 108]

##### 3.6.1.2 Category 1.2: Practitioner–Patient Relationship

The relationship between an Ayurvedic practitioner and their patient was based on trust, respect, and a common goal of health and well‐being [27]. In explanation, Ayurvedic practitioners emphasized the significance of the mutual respect that should have existed between the practitioner and the patient, whereby the patient was empowered to take responsibility for their health, whilst benefiting from the guidance of an expert [26]. They further insisted that Ayurvedic therapies could not work without listening to and understanding the patients' preferences. [26, 27]

“The practitioner and client were partners in the examination process. The practitioner was clearly in control of the knowledge but led the discussion in such a way that the client freely expressed his or her views, doubts, and realisations. The client was deeply involved in the entire process; such involvement may translate into greater compliance with the treatment plan.”

[26, pp. 132–133]

##### 3.6.1.3 Category 1.3: The Holistic Nature of Ayurveda

According to Ayurvedic practitioners, Ayurveda's holistic approach transcended conventional Western medical paradigms [25, 26]. They described that the holistic approach took into account the entire individual, including the person's body constitution (Prakruti), their current state of imbalances (Vikruti), their digestion (Agni), their diet (Aahara), and their daily practices (Dinacharya). Explaining that these factors all influenced health and well‐being [25, 26, 27]. One practitioner elaborated on this holistic perspective:

“This holistic view of the body is distinctive and contrasts somewhat with the biomedical focus on individual organ systems. The therapeutic framework for Ayurvedic supportive care after cancer treatment focuses on restoring equilibrium, building mental and physical strength, and rejuvenation.”

[15, p. 368]

##### 3.6.1.4 Category 1.4: Ayurveda's Role in Chronic Care

Ayurvedic practitioners explained that Ayurveda is commonly recognized for its role in chronic care [25]. Ayurvedic practitioners distinguished between the strengths of Ayurveda and Western medicine, noting that while Western medicine excels in handling medical emergencies and surgical interventions, Ayurveda is particularly suited for the long‐term management of chronic conditions [25, 26]. Ayurveda's ability to provide long‐term management for chronic conditions was attributed to its understanding of the true etiology of the disease, which made it a preferred choice for NCD management [26]. In one study, a practitioner explained this role:

“Ayurveda was known for rheumatic diseases, mental diseases, musculoskeletal diseases, and, in general, chronic diseases …. Ayurveda was always described as providing long‐term cures, especially for the so‐called chronic diseases—the reason being that it could determine the ‘true’ etiology of the disease.”

[25, p. 67]

##### 3.6.1.5 Category 1.5: Individualized Treatment Plans

Ayurvedic practitioners emphasized the individualized nature of their treatment plans. Unlike conventional Western one‐size‐fits‐all approaches, Ayurveda acknowledged and embraced the inherent uniqueness of each individual [26]. Ayurvedic practitioners achieved this by assessing a person's fundamental constitution (Prakriti) and current imbalances (Vikriti). Ayurvedic practitioners tailored treatment plans according to the individual needs and characteristics of each patient. This individualized approach aligned seamlessly with the contemporary shift toward patient‐centered care [14, 27]. In one study, a practitioner explained this approach as follows:

“Ayurvedic medicine treats the individual with reference to the Prakruti/Vikruti paradigm; therefore, the treatment is customized and individualised. Conventional medicine develops treatment based on treating large populations where formulations are standardised.”

[26, pp. 145–146]

#### 3.6.2 Synthesized Finding 2: Factors Influencing the Provision of Quality Ayurvedic Care

This synthesized finding described the diverse factors influencing the provision of quality Ayurvedic care. Some of these challenges included the challenges with integration, lack of access to Ayurvedic medicines, restrictions in practice, and lack of regulations. Addressing these issues ensured that individuals received high‐quality care from qualified Ayurvedic practitioners. This synthesized finding was derived from 25 findings that were merged into 4 categories (see Appendix SC).

##### 3.6.2.1 Category 2.1: Integration of Services

Ayurvedic practitioners recognized the challenges of integrating Ayurveda into conventional Western healthcare systems [27]. The challenge went beyond treatment integration, as it involved reconciling the differing methodologies and worldviews of each system [27]. While Western medicine prioritized empirical, evidence‐based practices, Ayurveda emphasized a holistic approach, using natural remedies and traditional healing practices [14]. In a study, one practitioner emphasized this need for integration:

“Every science has its own advantages and disadvantages and limitations. So, if they work hand in hand, like in the same clinic, so have a Western doctor and Ayurvedic doctor. They can discover a client's condition, and they can do things with an integrated approach. It's going to be very helpful for the client. So, actually, if you really have an aim to serve the humanity… they should work together.”

[14, p. 98]

Ayurvedic practitioners reported that overcoming these challenges required interdisciplinary education, standardized protocols, increased public awareness, and the creation of collaborative healthcare centers [14, 27].

##### 3.6.2.2 Category 2.2: Lack of Access to Ayurvedic Medicines

Ayurvedic practitioners in OECD countries reported encountering major challenges, with limited access to Ayurvedic medicine [25, 27]. The highly regulated framework around the import, production, and distribution of these medicines represented significant barriers to the supply chain for Ayurvedic medicines [25]. Ayurvedic practitioners reported challenges with selecting the proper herbs and negotiating the limited choices available to them [27].

“Here, as a practitioner, I've found that very difficult. Still, I'm finding nowadays it's more difficult… I have to think how the patient is going to get the maximum benefit… selecting the herbs which are available here, and that can give a maximum benefit to the patient… obviously limited—really limited—medication you can give out.”

[27, p. 148]

Despite these challenges, Ayurvedic practitioners remained determined to work with the available resources and emphasized the need to address these regulatory barriers to ensure broad access to Ayurvedic treatments [25, 27].

##### 3.6.2.3 Category 2.3: Restrictions in Practice and Lack of Regulation

Ayurvedic practitioners reported challenges in navigating governmental policies [25, 27]. For example, manufacturing therapeutic herbal products without Traditional Herbal Registration was illegal [25], but the licencing process was so expensive that many herbal remedies could not be licenced [27]. Ayurvedic practitioners also expressed concerns that the lack of regulation allowed untrained individuals to establish Ayurvedic clinics, leading to substandard or harmful treatments [25, 27]. In a study, one practitioner highlighted these concerns:

“The focus needs to shift now… Although Ayurveda is seemingly expanding, it is not necessarily good because of the lack of regulation … anyone can currently set up.”

[27, p. 103]

In this regard, Ayurvedic practitioners' welcome comprehensive regulations such as mandatory licencing, certification, and accreditation to allow only properly trained professionals to offer Ayurvedic treatments and protect the credibility of the practice [25, 27].

##### 3.6.2.4 Category 2.4: Improving the Ayurvedic Research Evidence Base

Some Ayurvedic practitioners emphasized the need for scientific research to support the acceptance and practice of Ayurveda, while others asserted that its success over centuries should have been enough for its acceptance [27]. Ayurvedic practitioners highlighted important gaps in the research, such as the need for scientific validation and standardization of treatments and conducting rigorous clinical trials that meet Western standards [25, 27]. They also stressed the importance of sharing findings in peer‐reviewed journals to validate Ayurvedic treatments [25, 27]. In a study, one practitioner emphasized the importance of research:

“I think research is very important, particularly because the whole thing is now, no matter if you say it's been working for thousands of years, but research has to provide the evidence. Without the evidence… modern medicine is not going to accept it.”

[27, p. 117]

## 4 Discussion

### 4.1 Summary of the Main Findings

This systematic review synthesized qualitative evidence on the experiences, perceptions, and perspectives of patients with NCDs and Ayurvedic practitioners regarding the use of Ayurveda for NCD management in OECD countries. Overall, of the nine included studies, six focused on exploring the experiences, perceptions, and perspectives of Ayurvedic practitioners [14, 15, 25, 27, 28, 29], and only two included studies focused on patients [12, 13]. This systematic review provided insights into some of the reasons patients with NCDs chose to use Ayurveda to manage their conditions, such as the perceived adverse effects of conventional Western medical treatment and patient dissatisfaction with prior Western medical treatment outcomes [12, 13]. These findings were consistent with those of previous systematic reviews on the reasons people chose to use complementary and alternative medicine [8, 31, 32, 33, 34, 35, 36]. This systematic review demonstrated that the Ayurvedic approach to the management of NCDs focuses on addressing the underlying causes of illness instead of managing symptoms alone. This approach could provide a sustainable and comprehensive solution to managing NCDs that lie outside of the conventional Western medicine predominantly symptom‐driven approach [12, 13]. This more root‐cause‐focused approach explains much of its appeal to those seeking a more integrative, upstream, person‐centered approach to managing NCDs [14, 15, 25, 27, 28, 29]. Additionally, this review demonstrated that the apparent holism in Ayurvedic practice had a strong appeal to some patients at a time when the reductionist philosophy underlying conventional Western medicine and NCD care was considered restrictive uninformative and unsatisfying [14, 15, 25, 26, 27, 28, 29]. These findings highlighted the need for discussions in Western medicine about integrating holistic and root‐cause‐focused approaches to NCD care. Such discussions could help develop more sustainable and practical healthcare models within Western medical practice [37, 38, 39, 40].

Despite Ayurveda's strengths, challenges in service delivery persisted. The limited availability of Ayurvedic medicines, particularly certain herbs and ingredients, posed a challenge and raised concerns about effective treatment [25, 27]. Ayurvedic practitioners in OECD countries reported difficulties in obtaining traditional herbs, which are often native to specific regions, making cultivation challenging. Additionally, restrictions in practice such as restrictions on the prescription of certain Ayurvedic herbs and the legal framework surrounding their use further impeded service delivery [25, 27]. The lack of regulation of Ayurveda in some OECD countries also raised concerns about the future of Ayurveda, this lack of regulation is seen as hindering the growth and credibility of Ayurvedic practice, prompting calls for comprehensive regulation to enhance accessibility and credibility [25, 27]. Collaboration emerged as a pivotal strategy for enhancing accessibility, Ayurvedic practitioners expressed a desire for collaboration with conventional Western practitioners, and the potential benefits of such collaboration are recognized as essential for providing patients with comprehensive and complementary care [30, 39]. Encouraging collaboration between Ayurvedic practitioners and Western medical professionals is also vital for an integrated healthcare approach [14, 15, 25, 26, 27, 28, 29]. However, divergent philosophies and paradigms between Ayurveda and Western medicine hindered seamless integration [26, 27]. Bridging the gap between Ayurveda and conventional Western medicine required open communication and mutual understanding between practitioners from both systems [14, 27].

### 4.2 Strengths and Limitations

To the best of our knowledge, this was the first qualitative systematic review that synthesized the experiences, perceptions, and perspectives of Ayurvedic practitioners and patients with NCDs regarding the use of Ayurveda for the management of these conditions. The inclusion of both patient and practitioner perspectives was a strength of this review and provided insight into Ayurveda's role in NCD management. The diversity of findings from OECD countries enhanced generalizability, offering insights into Ayurveda's use in regions where it was less commonly practised. This review adhered to JBI's rigorous methods, employed a comprehensive search strategy, and involved independent reviewers at each stage of the review. One of the potential limitations was the publication bias; there was a well‐known preference for research from certain countries and certain languages, predominantly English and Western medicine. Such bias marginalizes non‐Western medical systems such as Ayurveda, thereby restricting the diversity of medical knowledge and resulting in poor visibility in the academic literature. Another limitation was that the studies were from 2002 to 2016, so the current situation might have been different. Three non‐peer‐reviewed studies were included in this review, as these presented further insights that were not captured in other peer‐reviewed studies. They were nevertheless critically evaluated and did not impact credibility. Reporting of Ayurvedic practitioners' characteristics varied, with age, sex, qualifications, and years of NCD experience often unreported. The lack of this detailed information reduced transparency and limited the comparability and generalizability of the findings. Future studies should emphasize the comprehensive reporting of practitioner characteristics to improve validity and applicability.

### 4.3 Implications for Practice and Research

This review recommends future research in a wide variety of geographical, sociodemographic, cultural, and healthcare environments and contexts. Ayurvedic treatment plans for NCD patients should be administered by trained and qualified Ayurvedic practitioners in a safe and supportive environment. Promoting regulatory reforms such as standardized training, accreditation, and quality control, as well as increasing access to Ayurvedic treatments through financial support and insurance coverage, will be critical to ensuring the effective integration of Ayurveda into mainstream healthcare. Additionally, conducting robust clinical trials and fostering collaboration between Ayurvedic and Western medical practitioners will help validate Ayurveda's effectiveness and support its future role in NCD care.

## 5 Conclusions

Patients preferred Ayurveda because of its natural approach and fewer side effects, whereas Ayurvedic practitioners valued its holistic approach. However, its wider acceptance has been hampered by hurdles such as regulatory barriers and limited access to medicines. Strategies to overcome some of the barriers identified in this review as well as to promote the strengths discussed in this review may facilitate the effective use of Ayurveda to manage NCDs in OECD countries.

## Author Contributions

**Patricia Egwumba:** conceptualization, investigation, formal analysis, methodology, validation, writing – original draft, writing – review and editing. **Haiquan Wang:** formal analysis, methodology, validation, writing – review and editing, investigation. **Laura Nellums:** supervision, investigation, methodology, validation, writing – review and editing. **Manpreet Bains:** supervision, investigation, methodology, validation, writing – review and editing, formal analysis. **Kaushik Chattopadhyay:** conceptualization, supervision, investigation, formal analysis, methodology, validation, writing – review and editing. All authors have read and approved the final version of the manuscript. Patricia Egwumba had full access to all of the data in this study and takes complete responsibility for the integrity of the data and the accuracy of the data analysis.

## Ethics Statement

The authors have nothing to report.

## Consent

The authors have nothing to report.

## Conflicts of Interest

The authors declare no conflicts of interest.

## Transparency Statement

The lead author Patricia Egwumba affirms that this manuscript is an honest, accurate, and transparent account of the study being reported; that no important aspects of the study have been omitted; and that any discrepancies from the study as planned (and, if relevant, registered) have been explained.

## Supporting information

Feb 17 Appendix Manuscript ID HSR 2024 04 1100.

## Data Availability

This systematic review is based on publicly available data from studies that have been previously published and are accessible through academic databases and journal platforms. As no new primary data were generated or collected for this review, there are no additional data sets to share. All relevant data supporting the findings of this review are included within the published article and its Supporting Materials. Further details can be made available upon reasonable request to the corresponding author.
