# Supplementary material for: Ayurveda for Managing Noncommunicable Diseases in Organisation for Economic Cooperation and Development Nations: A Qualitative Systematic Review
Source: Health Sci Rep. 2025 Apr 8;8(4):e70624. doi: 10.1002/hsr2.70624 (PMC11976450; doi:10.1002/hsr2.70624)
Supplement: Supplementary file 1 — Feb 17 Appendix Manuscript ID HSR 2024 04 1100. [file HSR2-8-e70624-s001.docx]

**Appendix A: Search strategy**

**Published studies**

**MEDLINE (Ovid)**

1946 – 30^th^ January 2024; 2624 results

1 exp Australia/

2 exp Austria/

3 exp Belgium/

4 exp Canada/

5 exp Chile/

6 exp Colombia/

7 exp Costa Rica/

8 exp Czech Republic/

9 exp Denmark/

10 exp Estonia/

11 exp Finland/

12 exp France/

13 exp Germany/

14 exp Greece/

15 exp Hungary/

16 exp Iceland/

17 exp Ireland/

18 exp Israel/

19 exp Italy/

20 exp Japan/

21 exp Latvia/

22 exp Lithuania/

23 exp Luxembourg/

24 exp Netherlands/

25 exp New Zealand/

26 exp Norway/

27 exp Poland/

28 exp Slovakia/

29 exp Slovenia/

30 exp Spain/

31 exp Sweden/

32 exp Switzerland/

33 exp Turkey/

34 exp United Kingdom/

35 exp England/

36 exp Northern Ireland/

37 exp Scotland/

38 exp Wales/

39 exp United States/

40 exp "Democratic People's Republic of Korea"/

41 exp "Republic of Korea"/

42 exp Korea/

43 exp Mexico/

44 exp Portugal/

45 exp "Organisation for Economic Co-Operation and Development"/

46 (Australia or Austria or Belgium or Canada or Chile or Colombia or Costa Rica or Czech Republic or Denmark or Estonia or Finland or France or Germany or Greece or Hungary or Iceland or Ireland or Israel or Italy or Japan or Latvia or Lithuania or Luxembourg or Netherlands or Holland or New Zealand or Norway or Poland or Slovakia or Portugal or Slovenia or Spain or Sweden or Switzerland or Turkey or United Kingdom or UK or Britain or Great Britain or England or Northern Ireland or Scotland or Wales or United States or USA or United States of America or Republic of Korea or Democratic People's Republic of Korea or Korea or Mexico or OECD).mp.

47 1 or 2 or 3 or 4 or 5 or 6 or 7 or 8 or 9 or 10 or 11 or 12 or 13 or 14 or 15 or 16 or 17 or 18 or 19 or 20 or 21 or 22 or 23 or 24 or 25 or 26 or 27 or 28 or 29 or 30 or 31 or 32 or 33 or 34 or 35 or 36 or 37 or 38 or 39 or 40 or 41 or 42 or 43 or 44 or 45 or 46

48 exp Medicine, Ayurvedic/

49 Ayurved*.tw,ot.

50 *Medicine, Traditional/

51 exp Complementary medicine/tu [Therapeutic use]

52 ((plant* or herb* or medicin* or drug*or therap*or intervention*or extract* or formulation*or preparation* or supplement*) adj6 (Ayurved* or Hindu or Indian)).tw,ot.

53 exp Plants, Medicinal/tu [Therapeutic use]

54 exp Plant Extracts/tu [Therapeutic use]

55 exp Plants/tu [Therapeutic use]

56 ((plant* or herb*) adj6 (medicin* or drug* or therap* or intervention* or extract* or formulation* or preparation* or supplement*)).tw,ot.

57 exp Ethnobotany/

58 exp Ethnopharmacology/

59 (ethnobotan* or ethno botan* or ethnopharmacolog* or ethno pharmacolog*).tw,ot.

60 *Phytotherapy/

61 (phytotherap* or phyto therap*).tw,ot.

62 (Complementary and alternative medicine).mp.

63 CAM.mp.

64 48 or 49 or 50 or 51 or 52 or 53 or 54 or 55 or 56 or 57 or 58 or 59 or 60 or 61 or 62 or 63

65 exp Qualitative Research/

66 exp Interview/

67 exp Focus Groups/

68 exp Observation/

69 exp Case Reports/

70 exp Grounded Theory/

71 (qualitative* or interview* or focus group* or observations*).mp.

72 (Grounded Theory or Phenomenology* or Ethnograph* or Action research or Narrative research).mp.

73 (mixed method studies or qualitative systematic reviews).mp.

74 Documents.mp.

75 65 or 66 or 67 or 68 or 69 or 70 or 71 or 72 or 73 or 74

76 47 and 64 and 75

**Embase (Ovid)**

1974 – 30^th^ January 2024; 4786 results

1 exp Australia/

2 exp Austria/

3 exp Belgium/

4 exp Canada/

5 exp Chile/

6 exp Colombia/

7 exp Costa Rica/

8 exp Czech Republic/

9 exp Denmark/

10 exp Estonia/

11 exp Finland/

12 exp France/

13 exp Germany/

14 exp Greece/

15 exp Hungary/

16 exp Iceland/

17 exp Ireland/

18 exp Israel/

19 exp Italy/

20 exp Japan/

21 exp Latvia/

22 exp Lithuania/

23 exp Luxembourg/

24 exp Netherlands/

25 exp New Zealand/

26 exp Norway/

27 exp Poland/

28 exp Slovakia/

29 exp Slovenia/

30 exp Spain/

31 exp Sweden/

32 exp Switzerland/

33 exp United Kingdom/

34 exp England/

35 exp Northern Ireland/

36 exp Scotland/

37 exp Wales/

38 exp United States/

39 exp "Democratic People's Republic of Korea"/

40 exp "Republic of Korea"/

41 exp Korea/

42 exp Mexico/

43 exp Portugal/

44 exp "Turkey (republic)"/

45 exp "Organisation for Economic Co-operation and Development"/

46 (Australia or Austria or Belgium or Canada or Chile or Colombia or Costa Rica or Czech Republic or Denmark or Estonia or Finland or France or Germany or Greece or Hungary or Iceland or Ireland or Israel or Italy or Japan or Latvia or Lithuania or Luxembourg or Netherlands or Holland or New Zealand or Norway or Poland or Slovakia or Portugal or Slovenia or Spain or Sweden or Switzerland or Turkey or United Kingdom or UK or Britain or Great Britain or England or Northern Ireland or Scotland or Wales or United States or USA or United States of America or Republic of Korea or Democratic People's Republic of Korea or Korea or Mexico or OECD).mp.

47 1 or 2 or 3 or 4 or 5 or 6 or 7 or 8 or 9 or 10 or 11 or 12 or 13 or 14 or 15 or 16 or 17 or 18 or 19 or 20 or 21 or 22 or 23 or 24 or 25 or 26 or 27 or 28 or 29 or 30 or 31 or 32 or 33 or 34 or 35 or 36 or 37 or 38 or 39 or 40 or 41 or 42 or 43 or 44 or 45 or 46

48 exp Ayurveda/

49 Ayurved*.tw,ot.

50 *Medicine, Traditional/

51 exp alternative medicine/

52 ((plant* or herb* or medicin* or drug*or therap*or intervention*or extract* or formulation*or preparation* or supplement*) adj6 (Ayurved* or Hindu or Indian)).tw,ot.

53 exp medicinal plant/

54 exp plant extract/

55 ((plant* or herb*) adj6 (medicin* or drug* or therap* or intervention* or extract* or formulation* or preparation* or supplement*)).tw,ot.

56 exp ethnobotany/

57 exp ethnopharmacology/

58 (ethnobotan* or ethno botan* or ethnopharmacolog* or ethno pharmacolog*).tw,ot.

59 *Phytotherapy/

60 (phytotherap* or phyto therap*).tw,ot.

61 (Complementary and alternative medicine).mp.

62 CAM.mp.

63 48 or 49 or 50 or 51 or 52 or 53 or 54 or 55 or 56 or 57 or 58 or 59 or 60 or 61 or 62

64 exp qualitative research/

65 exp interview/

66 exp observation/

67 exp case report/

68 exp grounded theory/

69 (qualitative* or interview* or focus group* or observations*).mp.

70 (Grounded Theory or Phenomenology* or Ethnograph* or Action research or Narrative research).mp.

71 (mixed method studies or qualitative systematic reviews).mp.

72 Documents.mp.

73 64 or 65 or 66 or 67 or 68 or 69 or 70 or 71 or 72

74 47 and 63 and 73

**CINAHL (EBSCOhost)**

1937 – 30^th^ January 2024; 998 results

1. TX (Australia or Austria or Belgium or Canada or Chile or Colombia or Costa Rica or Czech Republic or Denmark or Estonia or Finland or France or Germany or Greece or Hungary or Iceland or Ireland or Israel or Italy or Japan or Latvia or Lithuania or Luxembourg or Netherlands or Holland or New Zealand or Norway or Poland or Slovakia or Portugal or Slovenia or Spain or Sweden or Switzerland or Turkey or United Kingdom or UK or Britain or Great Britain or England or Northern Ireland or Scotland or Wales or United States or USA or United States of America or Republic of Korea or Democratic People's Republic of Korea or Korea or Mexico or OECD)/ S1
2. (MH "Medicine, Ayurvedic")/ S2
3. TX (medicine, Ayurvedic or Ayurved*)/ S3
4. S2 or S3/ S4
5. (MH "Qualitative Studies+")/ S5
6. (MH "Interviews+")/ S6
7. (MH "Focus Groups")/ S7
8. TX (qualitative or interview* or focus group*)/ S8
9. TX (mixed method studies or qualitative systematic reviews)/ S9
10. (MH "Grounded Theory")/ S10
11. (MH "Phenomenology")/ S11
12. (MH "Ethnographic Research")/ S12
13. (MH "Action Research")/ S13
14. TX (Grounded Theory or Phenomenology* or Ethnographic research or Action research)/ S14
15. S5 or S6 or S7 or S8 or S9 or S10 or S11 or S12 or S13 or S14/ S15
16. S1 and S4 and S15

**PsycINFO (Ovid)**

1806 – 30^th^ January 2024; 3935 results

1 "Organisation for Economic Co-Operation and Development".mp.

2 (Australia or Austria or Belgium or Canada or Chile or Colombia or Costa Rica or Czech Republic or Denmark or Estonia or Finland or France or Germany or Greece or Hungary or Iceland or Ireland or Israel or Italy or Japan or Latvia or Lithuania or Luxembourg or Netherlands or Holland or New Zealand or Norway or Poland or Slovakia or Portugal or Slovenia or Spain or Sweden or Switzerland or Turkey or United Kingdom or UK or Britain or Great Britain or England or Northern Ireland or Scotland or Wales or United States or USA or United States of America or Republic of Korea or Democratic People's Republic of Korea or Korea or Mexico or OECD).mp.

3 1 or 2

4 exp "Medicinal Herbs and Plants"/

5 exp Alternative Medicine/

6 Ayurved*.tw,ot.

7 ((plant* or herb*) adj6 (medicin* or drug* or therap* or intervention* or extract* or formulation* or preparation* or supplement*)).tw,ot.

8 (ethnobotan* or ethno botan* or ethnopharmacolog* or ethno pharmacolog*).tw,ot.

9 (phytotherap* or phyto therap*).tw,ot.

10 (Complementary and alternative medicine).mp.

11 CAM.mp.

12 4 or 5 or 6 or 7 or 8 or 9 or 10 or 11

13 exp Qualitative Methods/

14 exp Interviews/

15 exp Focus Group/

16 exp Participant Observation/

17 exp Case Report/

18 exp Grounded Theory/

19 (qualitative* or interview* or focus group* or observations*).mp.

20 (Grounded Theory or Phenomenology* or Ethnograph* or Action research or Narrative research).mp.

21 (mixed method studies or qualitative systematic reviews).mp.

22 Documents.mp.

23 13 or 14 or 15 or 16 or 17 or 18 or 19 or 20 or 21 or 22

24 3 and 12 and 23

**AMED (Ovid)**

1985 – 30^th^ January 2024; 4372 results

1 exp Australia/

2 exp Canada/

3 exp Denmark/

4 exp Finland/

5 exp France/

6 exp Germany/

7 exp Hungary/

8 exp Iceland/

9 exp Ireland/

10 exp Israel/

11 exp Italy/

12 exp Japan/

13 exp Netherlands/

14 exp New Zealand/

15 exp Poland/

16 exp Spain/

17 exp Sweden/

18 exp Switzerland/

19 exp England/

20 exp Northern Ireland/

21 exp Scotland/

22 exp Wales/

23 exp United States/

24 exp Korea/

25 exp Mexico/

26 "Organisation for Economic Co-Operation and Development".mp.

27 (Australia or Austria or Belgium or Canada or Chile or Colombia or Costa Rica or Czech Republic or Denmark or Estonia or Finland or France or Germany or Greece or Hungary or Iceland or Ireland or Israel or Italy or Japan or Latvia or Lithuania or Luxembourg or Netherlands or Holland or New Zealand or Norway or Poland or Slovakia or Portugal or Slovenia or Spain or Sweden or Switzerland or Turkey or United Kingdom or UK or Britain or Great Britain or England or Northern Ireland or Scotland or Wales or United States or USA or United States of America or Republic of Korea or Democratic People's Republic of Korea or Korea or Mexico or OECD).mp.

28 1 or 2 or 3 or 4 or 5 or 6 or 7 or 8 or 9 or 10 or 11 or 12 or 13 or 14 or 15 or 16 or 17 or 18 or 19 or 20 or 21 or 22 or 23 or 24 or 25 or 26 or 27

29 exp Ayurvedic medicine/

30 exp Plants medicinal/

31 exp Plant extracts/

32 exp Ethnopharmacology/

33 (Complementary and alternative medicine).mp.

34 CAM.mp.

35 exp Traditional medicine/

36 exp Complementary medicine/

37 exp Complementary therapies/

38 29 or 30 or 31 or 32 or 33 or 34 or 35 or 36 or 37

39 exp Interviews/

40 exp Case report/

41 (qualitative* or interview* or focus group* or observations*).mp.

42 (Grounded Theory or Phenomenology* or Ethnograph* or Action research or Narrative research).mp.

43 (mixed method studies or qualitative systematic reviews).mp.

44 Documents.mp.

45 39 or 40 or 41 or 42 or 43 or 44

46 28 and 38 and 45

**Web of Science**

1900 –30^th^ January 2024; 729 results

#1 TI= (Australia or Austria or Belgium or Canada or Chile or Colombia or Costa Rica or Czech Republic or Denmark or Estonia or Finland or France or Germany or Greece or Hungary or Iceland or Ireland or Israel or Italy or Japan or Latvia or Lithuania or Luxembourg or Netherlands or Holland or New Zealand or Norway or Poland or Slovakia or Portugal or Slovenia or Spain or Sweden or Switzerland or Turkey or United Kingdom or UK or Britain or Great Britain or England or Northern Ireland or Scotland or Wales or United States or USA or United States of America or Republic of Korea or Democratic People's Republic of Korea or Korea or Mexico or OECD)

#2 TI= (“Ayurvedic medicine” or Ayurved* or “traditional medicine” or “complementary medicine” or ((plant* or herb* or medicin* or drug* or therap* or intervention* or extract* or formulation* or preparation* or supplement*) adj6 (Ayurved* or Hindu or Indian)) or “medicinal plants” or “plant extracts” or plants or ((plant* or herb*) adj6 (medicin* or drug* or therap* or intervention* or extract* or formulation* or preparation* or supplement*)) or ethnobotany or ethnopharmacology or (ethnobotan* or ethno botan* or ethnopharmacolog* or ethno pharmacolog*) or phytotherapy or (phytotherap* or phyto therap*))

#3 ALL= (Case reports or (Qualitative*or interview*or focus group*or observations*) or
(grounded theory or phenomenology* or ethnograph*) or (mixed method studies or qualitative systematic reviews) or documents)

#1 and #2 and #3

**Unpublished studies**

**EthOS**

9^th^ March 2023; 18 results

Ayurveda [any word] or Ayurvedic [any word]

**ProQuest Dissertations and Theses**

30^th^ January 2024; 1079 results

Ayurved* and summary((Australia or Austria or Belgium or Canada or Chile or Colombia or Costa Rica or Czech Republic or Denmark or Estonia or Finland or France or Germany or Greece or Hungary or Iceland or Ireland or Israel or Italy or Japan or Latvia or Lithuania or Luxembourg or Netherlands or Holland or New Zealand or Norway or Poland or Slovakia or Portugal or Slovenia or Spain or Sweden or Switzerland or Turkey or United Kingdom or UK or Britain or Great Britain or England or Northern Ireland or Scotland or Wales or United States or USA or United States of America or Republic of Korea or Democratic People's Republic of Korea or Korea or Mexico or OECD))

**Appendix B:**

**Table 1: Studies ineligible following full-text review**

| **Study** | **Reason for exclusion** |
| --- | --- |
| Freymann H, Rennie T, Bates I, Nebel S, Heinrich M. Knowledge and use of complementary and alternative medicine among British undergraduate pharmacy students. Pharm world sci. 2006;28(1):13-8. | Ineligible participants |
| Adams LL, Gatchel RJ, Gentry C. Complementary and alternative medicine: applications and implications for cognitive functioning in elderly populations. Altern Ther Health Med. 2001;7(2):52-61. | Ineligible participants |
| Shreffler-Grant J, Hill W, Weinert C, Nichols E, Ide B. Complementary therapy and older rural women: who uses it and who does not? Nurs Res. 2007;56(1):28-33. | Ineligible participants |
| Arcury T, Nguyen H, Sandberg J, Neiberg R, Altizer K, Bell R, et al. Use of Complementary therapies for health promotion among older adults. J Appl Gerontol. 2013;34. | Ineligible participants |
| Bremner M, Blake B, Stiles C. The experiences of persons living with HIV who participate in mind-body and energy therapies: a systematic review protocol of qualitative evidence. JBISRIR. 2015;13(10):41-9. | Ineligible participants |
| Chesney AP, Thompson BL, Guevara A, Vela A, Schottstaedt MF. Mexican American folk medicine: implications for the family physician. J Fam Pract 1980;11(4):567-74. | Ineligible phenomena of interest |
| Schmid K, Ivemeyer S, Vogl C, Klarer F, Meier B, Hamburger M, et al. Traditional use of herbal remedies in livestock by farmers in 3 Swiss cantons (Aargau, Zurich, Schaffhausen). Forsch Komplementmed. 2012;19(3):125-36 | Ineligible phenomena of interest |
| Rao S, Kini V, Hegde SK, Meera S, Rao P, George T, Ayurvedic Drug Triphala in combination with povidone-iodine mitigates radiation-induced mucositis in head and neck cancer patients without affecting the tumour response.Indian J Otolaryngol Head Neck Surg. 2023;75(3):1480-9. | Ineligible phenomena of interest |
| Naraindas H. Of relics, body parts and laser beams: the German Heilpraktiker and his Ayurvedic spa. Anthropol Med. 2011;18(1):67-86. | Ineligible phenomena of interest |
| Kim H, Song MJ. Analysis and recordings of orally transmitted knowledge about medicinal plants in the southern mountainous region of Korea. J Ethnopharmacol. 2011;134(3):676-96. | Ineligible phenomena of interest |
| Bautista-Cruz A, Arnaud-Vinas MR, Martinez-Gutierrez GA, Sanchez-Medina PS, Pacheco RP. The traditional medicinal and food uses of four plants in Oaxaca, Mexico. J Med Plant Res. 2011;5(15):3404-11. | Ineligible phenomena of interest |
| Rao D. Choice of medicine and hierarchy of resort to different health alternatives among Asian Indian migrants in a metropolitan city in the USA. Ethnicity & health. 2006;11(2):153-67. | Ineligible phenomena of interest |
| Antih J, Canigueral S, Heinrich M. Use of medicinal plants by the Bolivian community in the metropolitan region of Barcelona. Revista de Fitoterapia. 2016;16(2):141-52. | Ineligible phenomena of interest |
| Anderzen-Carlsson A, Persson Lundholm U, Kohn M, Westerdahl E. Medical yoga: another way of being in the world-a phenomenological study from the perspective of persons suffering from stress-related symptoms. Int. J. Qual. Stud. Health Well-being. 2014; 9:23033. | Ineligible phenomena of interest |
| Hamm E, Muramoto ML, Howerter A, Floden L, Govindarajan L. Use of provider-based complementary and alternative medicine by adult smokers in the United States: comparison from the 2002 and 2007 NHIS survey. AJHP. 2014;29(2):127-31. | Ineligible phenomena of interest |
| Liu L, Tang Y, Baxter GD, Yin H, Tumilty S. Complementary and alternative medicine - practice, attitudes, and knowledge among healthcare professionals in New Zealand: an integrative review. BMC Complement Med Ther. 2021;21(1):63. | Ineligible phenomena of interest |
| Kumari N. First and second-generation generic Indians on drug use: using cultural norms and yoga in drug prevention. Drugs Alcohol Today. 2004;4(4):21-9. | Ineligible phenomena of interest |
| Sjöstrand P, Lundh T, Skerfving S, Gustavsson P. Health food preparations have caused several cases of severe lead poisoning. At least four cases in Sweden after intake of an ayurvedic preparation. Lakartidningen. 2007;104(10):787-9 | Full-text not available |
| Dhruva A, Hecht F, Weaver J, Kaptchuk T, Lad V, Adler S. A whole systems approach to the study of Ayurveda for cancer survivorship: Results from a qualitative investigation. BMC Complement Altern Med. 2012;12(1). | Full-text not available |
| Adler SR, Hecht FM, Miaskowski C, Agarawal S, Kaptchuk TJ, Abrams DI, et al. Qualitative analyses from a prospective clinical study of a whole systems Ayurvedic intervention for breast cancer survivorship. J Altern Complement Med. 2014;20(5): A75-A. | Full-text not available |
| Creemers L, Van den Driessche M, Moens M, Van Olmen A, Verschaeren J, T'Syen M, et al. Safety of alternative medicines reconsidered: lead-induced anaemia caused by an indian ayurvedic formulation. Acta clinica Belgica. 2008;63(1):42-5. | Full-text not available |

**Appendix C: Synthesised findings**

**1: Synthesised findings based on Patients’ experiences, perceptions, and perspectives:**

**Table 2 Synthesised Finding 1: Reasons for Ayurvedic Use**

| Finding | Illustration | Category | ***SF1 -*Reasons for Ayurvedic Use (2 categories)**:  This synthesised finding was derived from 8 findings merged into 2 categories. This synthesised finding revealed that patients with NCDs chose to use Ayurveda mainly because of the perceived side effects and dissatisfaction with the efficacy of conventional Western medicines. Patients also reported using Ayurveda because it was a more natural option compared to Western medicine. |
| --- | --- | --- | --- |
| A more negative view was mainly due to concerns about the side effects of the frequent prescription of medication by biomedical practitioners. (U) | *“The reason why I sought Ayurveda was that I have ulcerous colitis, an inflamed bowel, which I have had for several years, and I’ve eaten medicine every day. I get a bit tired of just eating medicine.” ^12(p.7)^* | **Perceived side effects of conventional Western medicine (5 findings).**  This category combined 5 unequivocal findings. This category refers to the negative or unwanted effects that individuals believe they experience as a result of taking prescribed medications or undergoing specific medical treatments. |  |
| Concerns about the unnaturalness of biomedical medication. (U) | *“What scares you about taking sleeping pills? A: Well, because it would be so scary not to be able to sleep by myself, which is totally natural ... I mean, we are born to have daily rhythms and to in some ways have destroyed that ability, one’s own process would be very discomforting.” ^12(p.7)^* |  |  |
| Negative perceptions of biomedical drugs: side effects, limited efficacy. (U) | *“I used asthma sprays daily for half a year. Six months later, I felt terrible. I vomited day and night … Then I developed an oedema … I read the description of the spray’s eventual side effects, and there it was: vomiting and oedema. I thought I would lose it. I felt tricked because I had asked him three times: “Couldn’t it be because of the spray? … I never went to see him again. I told myself, 'You will not destroy me.' I had had enough.” ^13(p.228)^* |  |  |
| Concerned about the harmful effects of prescribed painkillers. (U) | *“The physician told me that I had rheumatism and gave me painkillers. A few days later, I saw on television how poisonous they were, and I confronted the physician, who said: 'There is nothing else.' That’s when I started looking for something else.” ^13(p.228)^* |  |  |
| Frustration and disappointment with painkillers. (U) | *“So, I called my family doctor and booked a time at once, and he told me to take painkillers. I took them for two weeks, but it didn’t help at all. Then the pain started spreading to all my muscles, I had pain in all my large muscles, arms, legs, Then I went to a doctor, and he didn’t know at all what it was. I went home from there just with more painkillers ... The doctor started examining me, they took a whole bunch of tests, but they couldn’t find anything!” ^12(p.7)^* |  |  |
| Lack of a holistic viewpoint in conventional medicine, mainly regarding environmental factors in illness causation (U) | *“As it was with my eczema, it was horrible, really all over my body. And there was no one [in biomedical healthcare] who asked me anything really. Not even what I worked with, in case there could be something in my work environment that I was reacting to. I mean there were no questions whatsoever, they were just ‘here you have some cortisone.” ^12(p.7)^* | **Dissatisfaction with previous conventional Western medical treatment outcomes (3 findings)**  This category combined 3 unequivocal findings. This category refers to the frustration patients feel when biomedical treatments fail to address the root causes of their health issues or provide holistic care. |  |
| Dissatisfaction with biomedical treatment's focus on pharmaceuticals, lack of lifestyle advice. (U) | *“My gastroenterologist didn’t help with nutrition. He only said: 'We have to try several drugs and see which one you tolerate.' But in my introductory conversation with my Ayurvedic physician, it became evident that Ayurveda offers much more, with advice that fits into my daily life.” ^13(p.236)^* |  |  |
| A disappointment with the lack of preventive measures in conventional medicine. (U) | *“But what I feel about Ayurveda is that it’s a way to develop oneself, both mentally and physically, to learn to listen to your body’s signals, to know what makes me healthy. It’s a way to keep myself healthy, which is something I feel western medicine doesn’t do whatsoever, but instead, there they just wait until you’re sick” ^12(p.7)^* |  |  |

**Table 3 Synthesised Finding 2: Perceived benefits and challenges of using Ayurveda**

| Finding | Illustration | Category | | ***SF2* Perceived benefits and challenges of using Ayurveda (2 categories):**  This synthesised finding was derived from 11 findings merged into 2 categories.  This synthesised finding elaborates on the perceived benefits of Ayurveda, such as Ayurveda’s effectiveness in relieving symptoms associated with NCDs. It also highlights the challenges expressed by patients such as adhering to complex Ayurvedic treatment regimens and the difficulty integrating it into their daily lives. |
| --- | --- | --- | --- | --- |
| Despite discontinuing her asthma medication, her health improved. (U) | *“I received a mixture because I have had asthma since I was small and have used an inhalator for many years. I have stopped using it now … During this period when I’ve used these herbs, I haven’t been ill.” ^12(p.9)^* | | **Relief of symptoms**  **(7 findings):**  This category combined 5 unequivocal and 2 credible findings. This category refers to how Ayurveda use leads to the alleviation or reduction of the discomfort of symptoms this involves decreasing the intensity or frequency of symptoms, improving overall well-being, and enhancing the quality of life |  |
| Cessation of rheumatic pains (U) | *“I had muscle rheumatism…[...] So I started doing as she said, I began with excluding everything ... and you know, within a week I was totally pain-free… Yes, I haven’t needed to eat medication since then!” ^12(p.9)^* | |  |  |
| Reduction in dependence on biomedical drugs. (U) | *“I managed to get through last summer without any cortisone, and I only needed 5% of my usual dose of asthma spray. I hadn’t changed my lifestyle, but the Ayurvedic drugs seemed to have an effect.” ^13(p.231-232)^* | |  |  |
| Thoughts as becoming more gathered and sleep improving (U) | *“I had more scattered thoughts before, but I sleep much better today and my thoughts are much more gathered.”^12(p.9)^* | |  |  |
| Found Ayurveda superior, more inclusive, holistic or individually accustomed than anything else they had tried out (C) | *“I have really tried to get help from all sorts of alternative medicine, and all types of treatment. [...]But for me, the major transformation came with Ayurveda.”^12^(p.10)* | |  |  |
| Improvement in overall health and immunity. (C) | *“Amazingly, since I started Ayurveda, I haven’t had a cold, a cough, any throat pain—nothing. Everyone has the flu, and I’m not susceptible. I think it has to do with Ayurveda." ^13(p.232)^* | |  |  |
| Management of joint pain without painkillers. (U) | *“After I started using sesame oil for my joints and changed my nutritional habits, I could already manage without any painkillers. Of course, I was not completely free of pain, but it was bearable.” ^13(p.232)^* | |  |  |
| The effects take a longer time to manifest (U) | *“The other advice I got, it’s like they say, that Ayurveda is a bit more long-term, that it’s not a quick fix that you do in a week. It’s about keeping at it for a long time. So I haven’t seen any clear effects from the advice yet.” ^12(p.9)^* | | **Challenges in using Ayurveda (4 findings):**  This category combined 3 unequivocal and 1 credible finding. This category refers to the difficulties patients encounter when integrating Ayurvedic practices into their lives, such as navigating complex treatment regimens, maintaining adherence to prescribed therapies, and overcoming limited understanding or knowledge of Ayurvedic principles. |  |
| Didn’t find the energy to follow the advice because of their health condition. (U) | *“The thing I felt, about this advice, is that it was a lot of things that I would have liked to follow, but I didn’t have the energy.” ^12(p.10)^* | |  |  |
| Indifference to Ayurvedic knowledge and limited interest in treatment methods. (C) | *“I knew nothing—only that it is a gentle medicine." ^13(p.236)^*  *"I only knew from hearsay that it was something natural, but I had no idea exactly what it was.”^13(p.236)^* | |  |  |
| Found the extent of advice unrealistic within the bounds of contemporary society (U) | *“But actually, it’s quite difficult these days, in this society, to apply everything that is recommended at a consultation.” ^12(p.9)^* | |  |  |

**2: Synthesised findings based on Ayurvedic practitioners’ experiences, perceptions, and perspectives:**

**Table 3 Synthesised Finding 1: Ayurvedic approach to NCD management**

| Finding | Illustration | | | Category | ***SF1 -* Ayurvedic approach to NCD management (5 categories):**  In this synthesised finding, 17 findings were aggregated into 5 categories. This synthesised finding explains Ayurveda’s fundamental principles and approach to NCD management. This finding explains how NCDs in Ayurveda are viewed as resulting from imbalances in the body's fundamental principles (doshas) and the accumulation of toxins (ama). |  |
| --- | --- | --- | --- | --- | --- | --- |
| Specific elements such as particular foods and hygiene products, cause emotional and physical ailments. (U) | *“Irregular food habits, junk food, and working for long hours, not sleeping during the night, and eating junk food in the middle of the night All those things, they will cause stress also. Result in the anxiety and everything.” ^14(p.94)^* | | | **Focus on treating the root cause. (3 findings)**  This category consisted of 2 unequivocal and 1 credible finding. This category highlights Ayurveda's central philosophy of addressing the underlying cause of illness rather than merely managing symptoms. |  |  |
| Problems typically originate from the patient’s environment, particularly exposure to chemicals, specific foods, and medications. (U) | *“It is the totality – environment. Many times people use so many deodorants, sprays this and that, which is going in your system ...” ^14(p.94)^* | | |  |  |  |
| Ayurveda focuses on treating the root cause of the imbalance. (C) | *“Allopathy suppresses symptoms and only offers standardised treatment options. On the other hand, Ayurvedic medicine treats the root cause and holistically treats the individual person through a gentler system, which encourages the body to heal itself.” ^26(p.108)^*  *“Fundamental differences between allopathy and Ayurveda. Ayurveda … is focused on treating the root cause of the imbalance. Conventional medicine, on the other hand, suppresses symptoms by external agents.”^26(p.145)^* | | |  |  |  |
| The client’s concerns were addressed throughout the session. (U) | | *“The practitioner and client were partners in the examination process. The practitioner was clearly in control of the knowledge but led the discussion in such a way that the client freely expressed his or her views, doubts, and realisations. The client was deeply involved in the entire process; such involvement may translate into greater compliance with the treatment plan." ^26(p.132-133)^* | | **Practitioner–patient relationship. (5 findings)**  5 unequivocal findings were merged to form this category. This category described the importance of a collaborative and trusting bond between Ayurvedic practitioners and their patients. In Ayurveda, this relationship is seen as crucial to effective treatment, as it allows for open communication and a deeper understanding of the patient’s physical, mental, and emotional state |  |  |
| Longer consultations compared to India, are seen as better for the practitioner-patient relationship. (U) | | *“But one thing which I observed. In India we normally use 10–15-minute consultations, but here normally it's a one-hour consultation. And we go detail about their daily routine and their diet, and what they take and everything we advise. That's the only difference.*  *Q - and do you think that's better or not...  - It's slightly better in one way. In one way it's better. The reason maybe, we get people who are really interested in Ayurveda." ^27(p.150)^* | |  |  |  |
| An egalitarian relationship allowed the practitioner and the patient to speak and listen to each other on an equal basis and agree on the treatment plan. (U) | | *“So, it's giving someone something to do that's within their capability, and that's why listening to them is important, like what they like to eat, so that you can just change it slightly or re-arrange the time and the pattern. Little things like that help.” ^27 (p.221)^* | |  |  |  |
| The practitioner took a substantial amount of time understanding the client’s history and concerns. (U) | | *"The history-taking portion probably took 1 hour... The practitioner communicated the details of their conversation to me later." ^26(p.133)^* | |  |  |  |
| The client was deeply involved throughout the whole process; such involvement may translate into greater compliance. (U) | | *"The practitioner and client had mutual respect ... She assured him that lifestyle changes along with herbs would bring positive changes in the body and mind." ^26(p.133)^* | |  |  |  |
| The consensus was that Ayurveda is a holistic system of healing that takes into account factors that manifest at all levels. (U) | | *“Ayurveda's obviously traditionally known as the Science of life. It's much more than - much more than medical science. I mean, it is regarded as medical science, but It's really knowledge of how to live; how to live healthily and happily, so It covers every dimension, physical, psychological and spiritual, so whatever can be conducive to a happy and healthy life. So that's why it's a kind of perfect, complete science.” ^27(p.94)^* | | **The holistic nature of Ayurveda (4 findings)** This category consisted of 2 unequivocal and 2 credible finding.  **This holistic Approach** emphasises treating the whole person by considering the interconnectedness of the body, mind, and spirit. It focuses on individualised care, addressing lifestyle, diet, and environmental factors to restore balance and prevent illness. This approach promotes long-term well-being and overall health |  |  |
| They also discussed the holistic approach of Ayurveda and the inter-connectedness of body systems. (U) | | *“This holistic view of the body is distinctive and contrasts somewhat with the biomedical focus on individual organ systems. The therapeutic framework for Ayurvedic supportive care after cancer treatment focuses on restoring equilibrium, building mental and physical strength, and rejuvenation.” ^15(p.368)^* | |  |  |  |
| Her previous efforts at contacting other holistic institutions such as spas, retreats, and ashrams ended in disappointment. (C) | | | *“Here, the problem I think is that they connect Ayurveda with spirituality. When I contacted some people by the phone [about Ayurveda], they said they are doing some yoga and meditation, and things like that. And they [asked] me if1know how to give classes on meditation - 1 said "No." "What about yoga?" And they don't want to know anything about Ayurveda ... they don't want pure Ayurveda; they want spirituality.” ^25(p.75)^* |  |  |  |
| Ayurveda’s holistic nature. (C) | | | “*What is Ayurveda? For me, Ayurveda is a typical science of life. You can live Ayurveda at any age, anywhere, whatever you're doing, and it really works... It works to balance yourself, to make you happy… Balance is not only the physical balance but the emotional and spiritual as well”^27 (p.96)^* |  |  |  |
| Most interviewees consulted both medical systems for specific diseases, depending on strengths and weaknesses. (U) | | | *“Ayurveda was known for rheumatic diseases, mental diseases, musculoskeletal diseases, and, in general, chronic diseases…. Ayurveda was always described as providing long-term cures, especially for the so-called chronic diseases - the reason being that it could determine the "true" aetiology of the disease. ^25(p.67)^* | **Ayurveda's role in chronic care. (3 findings)**  3 unequivocal findings constituted this category which emphasises Ayurveda’s effectiveness in managing long-term health conditions through personalised treatments. Ayurveda utilises herbal remedies, dietary adjustments, and lifestyle changes to promote balance and improve overall health. |  | |
| All her patients are fully committed to the Ayurvedic method, and all see improvement. (U) | | | *"She has seen improvement in her clients within 5 days of treatment." ^26(p.109)^* |  |  |  |
| Practitioner two acknowledged that allopathy is good for acute care and Ayurvedic medicine offers better chronic and preventative care (U) | | | *“Ayurvedic medicine also has specific remedies for acute care but they are difficult to practice in the United States since many involve heavy metal formulations ... Ayurvedic practitioners are not licensed to treat medical conditions, their role is limited to lifestyle consultations." ^26(p.104)^* |  |  |  |
| Customizing the medicines to each patient. (U) | | | *“We prescribe them herbal medications...powder, liquid, capsule form...it’s the customisation that counts." ^14(p.96)^* | **Individualised treatment plans (2 Findings)**  This category consisted of 1 unequivocal and 1 credible finding. **Individualized Treatment Plans** in Ayurveda focus on tailoring healthcare to each person's unique constitution, known as Prakruti. Ayurvedic practitioners consider various factors like body type, lifestyle, diet, and emotional state to create personalized treatment strategies. |  | |
| Ayurvedic treatment is customized and individualized. (C) | | | *“Ayurvedic medicine treats the individual with reference to the prakruti/vikruti paradigm; therefore, the treatment is customised and individualised. Conventional medicine develops treatment based on treating large populations where formulations are standardised.” ^26 (p.145-146)^* |  |  | |

**Table 4 Synthesised Finding 2: Factors Influencing** **the provision of quality Ayurvedic care**

| Finding | *Illustration* | | Category | ***SF2 Factors Influencing Ayurvedic Service Delivery (4 categories)***  This synthesised finding was created from 25 findings merged into 4 categories. This synthesised finding explains the various factors that impact Ayurvedic service delivery. These factors include the integration of services, lack of access to Ayurvedic medicines, restrictions in practise and lack of regulation. Addressing these issues will ensure that individuals have access to high-quality care from trained professionals in this ancient healing tradition. |
| --- | --- | --- | --- | --- |
| Referrals from Western healthcare practitioners. (U) | *"Mainstream doctors...are telling their patients nowadays to go and look for fellows who are practicing natural medicine..." ^14(p.97)^* | | ***Integration of services*. (10 findings)**  10 unequivocal findings were combined to create this category, which showed how Ayurvedic practitioners strongly advocate for the integration of Ayurvedic services into mainstream healthcare, seeing it as a way to enhance patient care through a more holistic approach. |  |
| Benefits of joint efforts between Western and Ayurvedic approaches. (U) | *“Every science has its own advantages and dis-advantages and limitations. So, if they work hand in hand, like in the same clinic, so have a Western doctor and Ayurvedic doctor. They can discover a client’s condition and they can do things with an integrated approach. It’s going to be very helpful for the client. So, actually, if you really have an aim to serve the humanity ... they should work together” ^14(p.98)^* | |  |  |
| Referrals to Western healthcare practitioners. (U) | *“For example, you come to me and tell me 'I have breast cancer'... I may have to confirm it by sending you for a mammogram." ^14(p.97-98)^* | |  |  |
| Increased acceptance of Ayurveda in the U.S. (U) | *"Practitioner one commented that with all the suppression by the medical establishment in the United States, it is a miracle that Ayurveda is gaining acceptance." ^26(p.100)^* | |  |  |
| A way forward is to work with GPs directly. (U) | *“My thinking was eventually to get the doctors to refer patients. ... I did do talks for doctors in Scotland and had quite a lot of success. 10 % of doctors attend talks on Ayurveda. Might lead to a huge response, lot of referrals and consultations. ^27(p.118-119)^* | |  |  |
| One area where most Ayurvedic practitioners agree is the ability of biomedicine to deal with situations which are either emergencies or high risk. (U) | *"There are so many aspects of modern medicine that are fantastic ... Surgery for a blocked artery ... It's unbelievable the technology." ^27(p.114)^* | |  |  |
| All 3 practitioners had positive comments on allopathic medicine and felt that in most cases it can complement Ayurveda. (U) | *“Practitioner Two acknowledged... Ayurveda is all-encompassing and open to other modalities... Practitioner One and Three commented that they have worked effectively with allopathic physicians as part of an integrative clinic. Practitioner Three added that she does not hesitate to refer her clients to other healers including* allopathic physicians and CAM practitioners.” *^26(p.144-145)^* | |  |  |
| Strong belief in the benefits of collaborative efforts between Western and Ayurvedic practices. (U) | *“So then we say that we can help you with the conjugative approach of Western and Eastern together at the official level, shorten the length of the waiting period, as well as lessen the burden on the hospitals.” ^14(p.98)^* | |  |  |
| Referrals working in both directions between Ayurvedic and Western practitioners. (U) | *“He [a South Asian medical doctor] used to refer clients to us...I used to give my clients to him, and he sent clients to us." ^14(p.97)^* | |  |  |
| Hope for an integrated healthcare system in which they work alongside biomedical practitioners. (U) | *"Q - But, at the moment, do you think there's a chance of Ayurveda integrating with modern medical? Thelma - I do, absolutely, yes. I just think It needs to be a little bit more understood and practised, and people getting good results from it." ^27(p.114)^* | |  |  |
| Ayurvedic consultations at Ayurvedic institutions in North America are too expensive and not as effective as in India. (C) | *“She related to me that most Canadians are not able to afford Ayurvedic treatment because other than common items that can be bought in Indian grocery stores, serious Ayurvedic treatment is only possible in India and most people cannot afford trips there.” ^25(p74)^* | | ***Lack of access to Ayurvedic medicines*. (7 findings)**  This category consisted of 4 unequivocal and 3 credible findings, emphasising the Lack of access to Ayurvedic medicines. Practitioners highlighted various barriers that impede the widespread availability of Ayurvedic treatments. |  |
| Lack of facilities and herbs as restrictions in Canada. (C) | *“Her main goal is to find work; she is frustrated because of the restrictions on her in Canada, such as a lack of facilities and herbs* but she is willing to work with whatever is available*.” ^25(p74)^* | |  |  |
| Practitioners described resorting to using remedies which have a reduced number of herbal components. (U) | *"The motto I have, because of all the things going on around here - you cannot prescribe this herb, or you cannot prescribe that herb, you keep on going and checking it and I Just have decided that I give more of the simple herbs ...” ^27(p.133)^* | |  |  |
| Due to restrictions, some practitioners discussed the possibility of making increased referrals to India. (U) | *“So, instead of following the formulae, you have to compromise because you are only allowed to prescribe what's there. I mean, I might recommend, if people really did need something quite serious, that they consider going to India." ^27(p.108)^* | |  |  |
| Practitioners cannot rely on learnt information from the textbooks as the remedies are unavailable and full treatments are not possible. (U) | *“Here, as a practitioner, I've found that very difficult. Still, I'm finding nowadays it's more difficult... I have to think how the patient is going to get the maximum benefit... selecting the herbs which are available here, and that can give a maximum benefit to the patient... obviously limited - really limited - medication you can give out.”​ ^27(p.148)^* | |  |  |
| Some practitioners are taking advantage of herbs that are common to both the Western and Ayurvedic pharmacopeia. (U) | *“ And that's why my practice probably Is slightly different from many people here because I use Ayurvedlc herbs as western herbs. I mean, their preparation is western. I use many tinctures. I don't use Ayurvedic classical formally apart from triphala. I don't stock any of those, so I don't give any of these classical medicines. I've never used them. I use guggul a lot but I use it as a tincture." ^27(p.140)^* | |  |  |
| 'Pure' Ayurvedic treatments are expensive. (C) | *"In her opinion, it is too expensive here to offer 'pure' Ayurvedic treatments, and there is not a wide-enough clientele..." ^25(p 75)^* | |  |  |
| Restricted by governmental and legal policies. (C) | *“He reasoned that he is restricted by governmental and legal policies as well as by the New Age demand for New Age products and services, which are only vaguely related to the Ayurveda he was trained in.” ^25(p 76)^* | | ***Restrictions in practice and Lack of Regulation* (4 findings)**  2 unequivocal and 2 credible findings were aggregated to create this category, which refers to the challenges Ayurvedic practitioners face due to limited regulatory frameworks and legal constraints in many regions. This category highlights the lack of standardised guidelines governing the practice of Ayurveda, leading to inconsistencies in the quality and safety of treatments. |  |
| Practice of Ayurveda in North America is different from Ayurveda in South India. (C) | *“She does not consider working for a New Age Ayurvedic spa or retreat to be practising the type of Ayurveda for which she was trained, but she does concede that such organisations have had the positive effect of bringing some ideas from Ayurveda into North American consciousness and sparking further interest in the subject.” ^25(p 73)^* | |  |  |
| Although Ayurveda may be growing, which would appear to be a good sign for the profession, the concerns over lack of regulation are also growing. (U) | *“The focus needs to shift now…Although Ayurveda is seemingly expanding, it is not necessarily good because of the lack of regulation…anyone can currently set up.”*  *​​^27 (p.103)^* | |  |  |
| Need for robust statutory regulation. (U) | *“ I feel, unless the profession is regulated, regulated quickly, we are doomed .... In [name of town) alone about 20 or 30 clinics have come up and we have received so many applications from people who are not properly qualified.” … Aarti - No. no. Some of them just 6 months training.”^27 (p.103)^* | |  |  |
| Dramatic changes in the public’s awareness of Ayurveda in America. (U) | | *"In the past, people did not accept herbs or changes to diet and lifestyle. Now, patients accept most aspects of Ayurvedic philosophy." ^26(p.99-100)^* | ***Improving the Ayurvedic research evidence base* (4 findings)**  4 unequivocal findings were grouped to create this category which focuses on the need to strengthen the scientific foundation of Ayurvedic practices through rigorous research. Ayurvedic practitioners and researchers emphasise the importance of conducting well-designed studies that align with modern scientific standards to validate the efficacy and safety of Ayurvedic treatments. |  |
| By-passing research. (U) | | *“I have helped several GPs with their own problems and they get converted. They are happy to try those things. They are actually phoning and asking us for help. I think the more we interact with western medical professionals and the more evidence they see, not Just research, It's personal Interaction...” ^27 (p.119)^* |  |  |
| Some practitioners in this study felt that research is necessary to convince the 'non-believer’ scientists. (U) | | *“Aarti - I think research is very important, particularly because the whole thing is now, no matter if you say it's been working for thousands of years, but research has to provide the evidence... modern medicine is not going to accept it. So we have to. Yes it's a pity, but we have to do it." ^27(p.117)^* |  |  |
| Participants felt that research assures the quality standards of herbs. (U) | | *"Research is important to identify the proper herbs, the potency of the herbs, and the quality control." ^27(p.116)^* |  |  |
